# The role of supportive supervision on immunization program outcome - a randomized field trial from Georgia

**DOI:** 10.1186/1472-698X-9-S1-S11

**Published:** 2009-10-14

**Authors:** Mamuka Djibuti, George Gotsadze, Akaki Zoidze, George Mataradze, Laura C Esmail, Jillian Clare Kohler

**Affiliations:** 1 Curatio International Foundation, Chavchavadze Avenue, 0162 Tbilisi, Georgia; 2 Tbilisi State Medical University, Pshavela Ave, Tbilisi; 3 Curatio International Consulting, Chavchavadze Avenue, 0162 Tbilisi, Georgia; 4 Leslie Dan Faculty of Pharmacy, Russel Street, University of Toronto, Toronto, ON, M5S 2S2, Canada

## Abstract

**Background:**

One of the most common barriers to improving immunization coverage rates is human resources and its management. In the Republic of Georgia, a country where widespread health care reforms have taken place over the last decade, an intervention was recently implemented to strengthen performance of immunization programs. A range of measures were taken to ensure that immunization managers carry out their activities effectively through direct, personal contact on a regular basis to guide, support and assist designated health care facility staff to become more competent in their immunization work. The aim of this study was to document the effects of "supportive" supervision on the performance of the immunization program at the district(s) level in Georgia.

**Methods:**

A pre-post experimental research design is used for the quantitative evaluation. Data come from baseline and follow-up surveys of health care providers and immunization managers in 15 intervention and 15 control districts. These data were supplemented by focus group discussions amongst Centre of Public Health and health facility staff.

**Results:**

The results of the study suggest that the intervention package resulted in a number of expected improvements. Among immunization managers, the intervention independently contributed to improved knowledge of supportive supervision, and helped remove self-perceived barriers to supportive supervision such as availability of resources to supervisors, lack of a clear format for providing supportive supervision, and lack of recognition among providers of the importance of supportive supervision. The intervention independently contributed to relative improvements in district-level service delivery outcomes such as vaccine wastage factors and the DPT-3 immunization coverage rate. The clear positive improvement in all service delivery outcomes across both the intervention and control districts can be attributed to an overall improvement in the Georgian population's access to health care.

**Conclusion:**

Provider-based interventions such as supportive supervision can have independent positive effects on immunization program indicators. Thus, it is recommended to implement supportive supervision within the framework of national immunization programs in Georgia and other countries in transition with similar institutional arrangements for health services organization.

**Abstract in Russian:**

See the full article online for a translation of this abstract in Russian.

## Abstract in Russian

See Additional file 1 for a translation of the abstract to this article in Russian.

## Background

A lack of experienced and quality human resources can easily jeopardize the success of any health program, including an immunization program. Alongside many variables that can cause poor coverage, such as inadequate financing, poor vaccine quality, poor vaccination practices, and weak health care systems [1], one of the most common barriers to improving immunization coverage rates is human resources and its management [2].

Effective human resource management has been defined as fundamental principle of quality health system performance [3]. To meet patients' needs in terms of provision of quality health services, the role of the workforce should be clearly defined, and it should be well deployed and organized [4]. In addition, a workforce must be motivated and appropriately skilled to do the job well [3].

Following independence, Georgia initiated widespread health care reforms in 1995. The reforms' key components included decentralization and reforming health care financing [5]. Recent reports suggest that the reforms conceived in 1995 were neither comprehensive enough nor well implemented [6]. The decentralization of health care financing and service supply responsibilities to the municipal level caused fragmentation and the delegation of powers was unclear and created unclear lines of responsibility [6]. These reforms affected much of the health care sector, including the National Immunization Program (NIP). Although Georgia has scaled up its vaccination coverage since 1995, coverage rates remained poor over the course of the reforms. For example, estimates in 2003 obtained from Georgia's new Immunization Management Information System (MIS) report coverage rates of 75% for DPT-3 and Polio-3, 48% for Hepatitis B-3 and 82% for Measles-1 [7]. While recognizing the role of many factors that can influence low coverage rates, an important factor which has caused a negative effect has been weak human resource management within the NIP, namely weak organizational structure and processes and a lack of knowledge and skills in management and supervision, especially at peripheral levels [7].

Overall, there is a limited amount of peer-reviewed medical literature addressing supportive supervision. Supportive supervision is defined here as a range of measures to ensure that personnel carry out their activities effectively through direct, personal contact on a regular basis to guide, support and assist designated staff to become more competent in their work. Two studies showed that nursing performance can benefit from supportive management and supervision [8,9]. The intervention was also shown to increase the health services efficiency (in terms of best use of resources) and equity (in terms of health care provision according to people's needs), achieving a substantial reduction of the burden of disease at reasonable cost [10].

Supervision of primary health care providers was tested through a randomized trial in Zimbabwe, which showed that, following supervision, overall drug management improved significantly compared with control and comparison groups. The study also showed that supervision can have a positive effect on improving performance in areas other than those supervised. Allocating resources to supervision is likely to result in improved performance of health workers with regard to the rational use of essential drugs, resulting in improved efficiency and effectiveness [11].

In developing countries, staff working in peripheral facilities where supervision is problematic deliver most primary health care services. A controlled field trial conducted in the Philippines examined whether systematic supervision using an objective set of indicators could improve health worker performance. In the intervention facilities, a correlation was found between frequency of supervision and improvements in performance. The authors concluded that systematic supervision using clearly defined and quantifiable indicators can improve service delivery considerably, at a modest cost [12]. One particular study documented that intense supervision led to high provider performance in systematic influenza and pneumococcal vaccination in a busy public emergency department setting despite initial resistance and extreme variation in individual performance [13].

Evidence obtained from the aforementioned studies suggest that promoting supportive supervision among managers of immunization programs may have a beneficial effect in transition countries (e.g. Georgia), where these programs are often impeded by factors such as poorly trained personnel and limited financial resources for health care workers.

Thus, the objective of the study was to document the effects of "supportive" supervision on the performance of the immunization program at the district(s) level in Georgia.

## Methods

### EPI service organization in the country

In Georgia there are 67 districts, and each district has a population of around 40,000-50,000 living in a district centre (small town) and surrounding 15-30 villages/communities. This unit (i.e. district) was chosen as the cluster unit of randomization. Normally, health workers working at district polyclinics (there is one such facility per district centre) and village ambulatories (usually one ambulatory per village) provide primary health care (including immunization) to district populations. The number of personnel available at primary health centres is determined by the size of the target population. Supervision of health workers for immunization work is done by a district immunization manager working out of the district Centre of Public Health (CPH). The immunization manager's job is to supervise, monitor and evaluate immunization programs, including vaccine supply/cold chain and operating district level immunization MIS. In every district, there is a CPH, which reports to the National Centre for Disease Control and Public Health, including on immunization work carried out within the framework of the NIP. In intervention districts, there were 194 health facilities and 778 primary healthcare workers (373 primary care doctors, and 405 primary care nurses) responsible for immunization, which were supervised by 31 immunization managers working in 15 district CPH.

### Design

The effectiveness of the intervention package was assessed through a pre-post experimental research design, supplemented with the qualitative data from focus group discussions (FGDs), where possible.

Stratified cluster randomization was used to select the 30 cluster units out of the nation's 67 districts and allocate them into the two study groups (intervention and control), yielding two allocation sequences of 15 clusters each. Baseline covariates used for stratification were immunization program performance indicators (DPT3 and HEPB3 coverage) for 2003. Intervention and control districts were assigned by random allocation with a table of random numbers.

A cohort of individuals responsible for immunization program management within district CPH and health care providers responsible for provision of immunization services to populations within the 15 districts selected served as the intervention group. Immunization program managers and providers within 15 control districts were selected to help validate any resulting changes in individual level outcome indicators within the intervention group. Measurements were assessed at the baseline and at the end of the one-year intervention on an individual level. Given that immunization managers supervise health workers only within their districts, and similarly health workers provide immunization services to target population residing in communities within the same district, the risk of contamination of the control group with the intervention is negligible. Use of smaller units (e.g. village) would have posed a higher risk of contamination of intervention activities in control clusters.

The Institutional Review Boards of Tbilisi State Medical University and the Office of Research Ethics, University of Toronto approved the protocol of the study. All participants gave informed written consent.

### Intervention

The overall intervention evaluated in this study consisted of a package of activities, which included development of supportive supervision guidelines for district immunization managers, district-level training in continuous supportive supervision, monitoring and evaluation of performance, and funding for district CPH to carry out the package of interventions (travel and communication costs). Supportive supervision, which was the focal point of the package of interventions, was based on a) introducing updated job descriptions with documented lines of supervision, b) improving communication lines and skills, and c) introducing guidelines and tools for supervision, performance review and monitoring, and evidence-based action planning, all of which help health workers to improve immunization service delivery.

Guidelines and tools for supervision included detailed instructions for conducting supervision, namely, sequence for conducting supervision meeting, checklist for supervisory visit, self assessment for providers, work planning action sheets, do's and don'ts of supervision, supervisor competencies, tips on delegation, tips on giving feedback, tips on resolving conflict, and tips on conducting difficult conversations. Every immunization manager from the intervention group was visiting each subordinated health facility at least once in a month.

Full-scale implementation of the project operations (supportive supervision) started in January 2005. Immunization managers from 15 intervention districts were trained to apply supportive supervision guidelines in practice. The subsequent 12 months involved extensive monitoring and on-the-job training of immunization managers and supervisors to improve supervision practices to help providers to solve problems related to immunization.

### Participants and sample size

Assuming a proportion of PHC providers having job descriptions of 44.4% in the control group, we calculated that a sample size of 300 PHC providers in each study group would be sufficient to detect at least 10% higher proportion of providers with updated job descriptions in the intervention group with 90% power and an unadjusted type-1 error of 5%. We also considered a potential non-response rate of 30% for both groups. The sample size was calculated for the comparison of two proportions. As for the number of cluster units, i.e. districts, we had to consider the budgetary limitations of the research project taking into account the considerable cost of implementing the intervention (funding for district CPH to carry out the package of intervention including travel and communication costs).

PHC providers were selected randomly to complete self-administered questionnaires at baseline and at follow up, thus two independent random samples were assembled. Baseline data were collected from 197 PHC providers in intervention districts and 195 PHC providers in control districts (overall response rate 65.3%). At follow-up, the number of respondents in intervention districts was 282 and 239 in control districts (overall response rate 86.8%).

In total, 15 immunization managers were selected randomly in both intervention and control group (one manager per district CPH) to complete the self-administered questionnaire at baseline (pre test). Out of these, 14 immunization managers from intervention districts and 12 immunization managers from control districts completed the questionnaires. This was followed by the training on supportive supervision attended by all 31 immunization managers working at intervention district CPH. At the follow up, self-administered post-test questionnaires were completed by all 31 immunization managers from intervention districts and by 15 immunization managers from control districts. Out of all 46 individuals that participated in at least one round, 26 immunization managers (14 from intervention districts and 12 from control districts) participated in both rounds of data collection.

### Data collection and management

#### Quantitative data

Outcome measures were ascertained using a self-administered questionnaire to a cohort of CPH immunization managers within intervention and control districts at baseline and again at follow-up. The questionnaire consisted of Likert scale, yes/no and open-ended questions. The questionnaire was pre-tested prior to data collection. Collected data were managed by MS Office Access database format.

#### Qualitative data

The survey was complemented by FGDs that were conducted pre- and post-intervention in December 2004 and February 2006 within intervention districts. In total, the following eight groups of individuals from intervention CPH offices and health care facilities were included in the FGDs:

District CPH office immunization managers (baseline and follow up)

District CPH office directors (baseline and follow up)

Health care facility manager (baseline and follow up)

Health provider (baseline and follow up)

For each group, the size of the group ranged from five to seven individuals. Focus group guides were developed separately for CPH staff and for health care providers. Participants were mostly the same individuals for the two rounds. The length of the discussion sessions averaged between two and two and a half hours for CPH staff and between one and one and a half hours for the facility staff. Two researchers conducted each focus group discussion and were joined by a moderator who led the discussion and a facilitator who handled logistics and took notes. The facilitator recorded the participant demographics, the time, duration, and location. As far as possible, the discussions took place in a setting where the session was not interrupted. Each of the FGDs were audiotaped and transcribed. The research team created a coding scheme using broad categories to organize the data, such as the overall performance of immunization program, the change in work environment, the barriers to supportive supervision, and perceived value of supportive supervision. Using these predefined codes, information was organized and displayed.

#### Outcome indicators

The following indicators were measured for study purposes:

- District level service delivery outcome indicators that helped to measure effectiveness of the intervention package pertained to: 1) immunization coverage; 2) rate of contraindications to vaccination (as diagnosed by health care providers); 3) rate of refusals to vaccination (as declared by parents); and 4) vaccine wastage.

- Individual-level outcome indicators pertained to: 1) perceived quality of organization of work at their CPH office/facility; 2) knowledge of how to carry out supportive supervision; 3) motivation/need to provide/receive supportive supervision; and 4) perceived barriers to implementing supportive supervision.

Five-point Likert scale questions, with responses ranging from strongly disagree to strongly agree, were used to ascertain information for constructing individual-level baseline and outcome indicators. The decision to focus on perceptions and motivation of district CPH and health care facility staff was based on the premise that improvements in motivation to practice supportive supervision and the knowledge and attitude towards supportive supervision are necessary to improve the performance of immunization program [14].

A univariate analysis was used to compare outcomes pre/post-test. A multivariate regression model was used to account for potential confounders. Qualitative data were analyzed using standard qualitative analysis methods [15].

#### Statistical methods

All statistical analyses were conducted using SPSS 12.0^©^. Differences between groups for categorical variables were assessed using Chi-square, and for continuous variables - analysis of variance (ANOVA). Differences between pre and post-test for continuous variables within the same group were assessed by paired-sample t-test. For all tests, a two-sided P-value < 0.05 was considered statistically significant.

To delineate an independent role of the intervention in improving individual level outcomes among immunization managers, we conducted multivariate analysis. Namely, the analysis of individual Likert scale outcomes was modelled using the Generalized Linear Model (GLM) for repeated measures. Indicators pertaining to individual level outcomes within intervention and control groups were measured among the same individuals at baseline and again one year later at follow-up after the implementation of the intervention. Age and years of experience in the current job were included to control for individual-level confounders. Geographic area was included to control for a number of differences that may exist between big cities and small towns/rural areas, hypothesized to include such potential confounders as governmental funding, access to health care, difficulties with transportation of immunization managers, etc. The interaction term "treatment group* geographic area" was also included in the model.

The independent role of the intervention in improving district level service delivery outcomes was assessed by comparing the treatment groups in terms of the changes from baseline, (i.e. magnitude of these changes ("change magnitude")). The difference between groups for the "change magnitude" was assessed using ANOVA.

## Results

### Demographic and employment characteristics

Demographic and employment characteristics were similar among CPH staff respondents in the intervention and control groups, both at baseline and follow up (Table 1). The majority of both intervention and control respondents were females, their mean age was slightly lower in the intervention districts compared with control districts, though with no statistical difference between groups. There was slight difference in the mean years in current job, again with no statistical difference between groups.

**Table 1:** Demographic and employment characteristics among immunization managers and service providers by treatment group and pre- and post-test.

Immunization managers	Treatment group	Pre-test (N = 14, 12)	Post-test (N = 31, 15)
1. Proportion of females	Intervention	80.0%	96.8%
	Control	80.0%	80.0%
	*X*^2^	*P = 0.674*	*P = 0.095*
			
2. Mean age (SD)	Intervention	41.1 (8.79)	42.3 (7.60)
	Control	44.5 (8.47)	46.9 (8.11)
	*ANOVA*	*P = 0.290*	*P = 0.068*
3. Mean years in current job (SD)	Intervention	4.1 (2.17)	5.8 (3.54)
	Control	5.5 (4.38)	7.2 (2.96)
	*ANOVA*	*P = 0.082*	*P = 0.185*
			

**Health providers**	**Treatment group**	**Pre-test (N = 197, 195)**	**Post-test (N = 282, 239)**

1. Proportion of females	Intervention	95.4%	93.3%
	Control	96.9%	95.0%
	*X*^2^	*P = 0.304*	*P = 0.262*
2. Mean age (SD)	Intervention	44.8 (9.51)	43.5 (9.67)
	Control	46.3 (9.34)	45.7 (9.70)
	*ANOVA*	*P = 0.112*	*P = 0.011*
			
3. Mean years in current profession, mean (SD)	Intervention	18.7 (10.18)	17.6 (10.13)
	Control	20.9 (10.06)	19.5 (10.52)
	*ANOVA*	*P = 0.027*	*P = 0.035*

SD, standard deviation.

The only statistically significant difference was that health providers in the control districts appeared to be older at post-test (45.71 *vs. *43.54 years, ANOVA, P = 0.011), and have longer experience in working in the current profession (at pre test: 20.98 *vs. *18.70 years, ANOVA, P = 0.027; at post test: 19.48 *vs. *17.57 years, ANOVA, P = 0.027) (Table 1).

### Evaluation results

Results of the survey demonstrate that the intervention package was implemented as intended within the 15 intervention districts. Table 2 shows that all immunization managers in intervention districts received supportive supervision guidelines. All but one supervisor (96.8%) were trained prior to the intervention on how to use supervision guidelines, execute performance reviews, and monitor achievements. In the control districts, none of the supervisors were trained in supportive supervision or received the supervisory guidelines. Supervisors in intervention districts visited subordinate health care facilities on average once a month, whereas supervisory visits in the control districts were at approximately once every two and a half months (ANOVA, p = 0.000). At baseline, the proportion of health providers having job descriptions was higher in the intervention districts compared with control districts and this difference was statistically significant (54.5% *vs. *44.4%, *X*^2^, p = 0.034). However, at follow up, this difference between the two groups was more profound (84.2% *vs. *49.8%, *X*^2^, p = 0.000).

**Table 2:** Details on implementation of intervention among immunization managers and health providers by treatment group and pre- and post-test.

Immunization managers	Treatment group	Pre-test (N = 14, 12)	Post-test (N = 31, 15)
1. Proportion of CPH offices receiving the supervisory guidelines to assist designated/supervisory staff to become more competent in their work	Intervention	0	100
	Control	0	0
	*X*^2^		***P = 0.000***
			
2. Proportion of CPH staff/supervisors trained how to use guidelines in supervision, performance review, and monitoring achievements	Intervention	0	96.8
	Control	0	0
	*X*^2^		***P = 0.000***
			
3. Average number of supervisory visits to each subordinated health care facility per month, mean (SD)	Intervention		1.00 (0.00)
	Control		0.37 (0.33)
	*ANOVA*		***P = 0.000***

**Health providers**	**Treatment group**	**Pre-test (N = 197, 195)**	**Post-test (N = 282, 239)**

1. Proportion of health facility staff having job descriptions with documented lines of supervision	Intervention	54.5	84.2
	Control	44.4	49.8
	*X*^2^	***P = 0.034***	***P = 0.000***

Table 3 presents individual level outcome indicators for both immunization managers and health providers. These indicators are calculated as mean values of Likert scale questions (1 = strongly disagree, 2 = disagree, 3 = neither agree nor disagree, 4 = agree, 5 = strongly agree). Immunization managers in both intervention and control districts were satisfied with organization of work in their CPH at baseline and follow up. In contrast, health providers were less satisfied with organization of work in their facility, however between baseline and follow up, no significant improvement was observed. Immunization managers in both intervention and control districts felt capable of carrying out supportive supervision both at baseline and follow up, and were persistent in declaring a high professional motivation to provide supportive supervision to subordinated staff. Likert scale scores were relatively lower for financial motivation at baseline, albeit with some increase from baseline to follow up, but still with no statistically significant difference between intervention and control groups. At follow up, immunization managers in intervention districts had significantly better perceived knowledge on how to carry out supportive supervision compared with immunization managers in control districts, and health providers in intervention districts were more confident that they were in need in supervision from immunization managers as compared with providers from control districts.

**Table 3:** Likert scale question on organization of work and supportive supervision among immunization managers and service providers by treatment group and pre- and post-test (values range from 5 = strongly agree to 1 = strongly disagree).

	Mean values and SD from Likert scale		
	
Immunization managers	Treatment group	Pre-test (N = 14, 12)	Post-test (N = 31,15)
1. I am satisfied with organization of work in their CPH	Intervention	3.60 (0.83)	3.87 (0.85)
	Control	3.87 (0.64)	3.60 (0.83)
	*ANOVA*	*P = 0.33*	*P = 0.31*
			
2. The overall work environment is good in our CPH	Intervention	3.47 (0.99)	3.58 (0.92)
	Control	3.20 (1.01)	3.40 (0.83)
	*ANOVA*	*P = 0.47*	*P = 0.52*
			
3. Feel capable of carrying out supportive supervision	Intervention	4.13 (0.35)	4.52 (0.51)
	Control	3.87 (0.64)	4.53 (0.52)
	*ANOVA*	*P = 0.17*	*P = 0.92*
			
4. Possess sufficient knowledge to carry out supportive supervision	Intervention	3.00 (0.75)	4.45 (0.62)
	Control	3.20 (0.77)	3.33 (1.05)
	*ANOVA*	*P = 0.48*	***P = 0.000***
			
5. Are professionally motivated to provide supportive supervision to subordinated staff on a regular basis	Intervention	4.20 (0.41)	4.45 (0.51)
	Control	4.13 (0.35)	4.27 (0.46)
	*ANOVA*	*P = 0.64*	*P = 0.24*
			
6. Are financially motivated to provide supportive supervision to subordinated staff on a regular basis	Intervention	2.40 (0.63)	3.32 (1.08)
	Control	2.27 (0.70)	3.47 (1.30)
	*ANOVA*	*P = 0.59*	*P = 0.69*

**Immunization service providers**	**Treatment group**	**Pre-test (N = 197, 195)**	**Post-test (N = 282, 239)**

1. Organization of work in their facility is not good	Intervention	2.56 (0.94)	2.24 (0.86)
	Control	2.38 (0.96)	2.33 (0.94)
	*ANOVA*	*P = 0.058*	*P = 0.27*
			
2. Feel they need supervision from immunization managers	Intervention	3.73 (0.90)	3.58 (0.83)
	Control	3.40 (0.98)	3.41 (0.95)
	*ANOVA*	***P = 0.01***	***P = 0.033***

Table 4 suggests that in intervention districts a number of barriers to implementing supportive supervision, as perceived by immunization managers, were removed or weakened over the course of intervention. These included: the "existence of a clear format for providing supportive supervision", "providers' recognition of the importance of supportive supervision", "the availability of resources to conduct supportive supervision", and "immunization managers' knowledge of how to conduct supportive supervision". A "lack of penalties for supervisors if providers' performance is low" was recognized as a barrier by immunization managers in both intervention and control districts and this perception did not change from baseline to follow up. A "lack of time to supervise facilities", and particularly a "lack of willingness to conduct supportive supervision" were not considered as barriers at both time points. Immunization managers in intervention districts, as compared with managers in control districts, have changed their perception that they are not paid enough to do supportive supervision, but the difference between the groups did not reach statistical significance at follow up. Health providers in both intervention and control districts did not agree on the lack of effective management and support from the upper level; however, respective Likert scale scores were significantly lower among providers in intervention districts as compared with that in providers from control districts.

**Table 4:** Likert scale question on main barriers to implementing supportive supervision among immunization managers and providers by treatment group and pre- and post-test (from 5 = strongly agree to 1 = strongly disagree).

	Mean values and SD from Likert scale		
	
Immunization managers	Treatment group	Pre-test (N = 14, 12)	Post-test (N = 31,15)
1. There is no clear format for providing supportive supervision to facilities and providers responsible for immunization	Intervention	3.87 (0.64)	2.21 (0.82)
	Control	3.73 (0.59)	3.27 (0.88)
	*ANOVA*	*P = 0.56*	***P = 0.000***
			
2. Providers do not recognize the importance of receiving supportive supervision	Intervention	3.53 (0.99)	2.14 (0.74)
	Control	3.00 (0.85)	2.87 (0.92)
	*ANOVA*	*P = 0.12*	***P = 0.007***
			
3. There is no penalty for supervisors if providers performance is low	Intervention	4.27 (0.46)	3.76 (0.99)
	Control	4.07 (0.46)	4.07 (0.46)
	*ANOVA*	*P = 0.24*	*P = 0.26*
			
4. Immunization managers do not have the time to supervise facilities/providers rendering immunization services to population	Intervention	2.27 (1.03)	2.28 (0.92)
	Control	2.40 (0.74)	2.33 (1.05)
	*ANOVA*	*P = 0.69*	*P = 0.85*
			
5. Immunization managers do not have resources to supervise facilities/providers rendering immunization services to population	Intervention	3.93 (0.88)	2.90 (1.05)
	Control	3.93 (0.46)	3.87 (0.83)
	*ANOVA*	*P = 1.00*	***P = 0.003***
			
6. Immunization managers do not know how to do supportive supervision	Intervention	3.67 (0.62)	1.83 (0.54)
	Control	3.80 (0.56)	3.87 (0.73)
	*ANOVA*	*P = 0.54*	***P = 0.000***
			
7. Immunization managers do not have the willingness to do supportive supervision	Intervention	1.93 (0.46)	1.79 (0.49)
	Control	2.00 (0.00)	1.80 (0.68)
	*ANOVA*	*P = 0.58*	*P = 0.97*
			
8. Immunization managers are not paid enough to do supportive supervision	Intervention	3.87 (0.74)	2.66 (1.05)
	Control	3.93 (0.79)	3.20 (0.77)
	*ANOVA*	*P = 0.82*	*P = 0.083*

**Health providers**	**Treatment group**	**Pre-test (N = 197, 195)**	**Post-test (N = 282, 239)**

1. There is no effective management and support from the upper level	Intervention	2.55 (1.02)	2.15 (0.77)
	Control	2.56 (0.98)	2.37 (0.89)
	*ANOVA*	*P = 0.94*	***P = 0.003***

Based on the results of multivariate analysis, the effect of the intervention among immunization managers was found to have independently contributed to the improvement of self-perceived knowledge to carry out supportive supervision (*p *= 0.034), as the mean score for this question increased by 1.09 among the intervention group while decreased by 0.14 among the control group pre and post-test (Table 5). The intervention also had a significant impact on decreasing the number of self-perceived barriers to supportive supervision including the knowledge of how to perform supportive supervision (P-value = 0.008), availability of resources to supervise immunization providers (P-value = 0.024), a lack of clear format for providing supportive supervision (P-value = 0.022), and a lack of recognition among providers on the importance of supportive supervision (P-value = 0.002).

**Table 5:** Results of regression analyses assessing the impact of the intervention on individual level outcome indicators for knowledge and use of supportive supervision among immunization managers.

	Mean values from Likert scale			Regression results			
		
Outcome indicator, n = 26 observations	Treatment group	Pre-test	Post-test	Model*	F statistic	P-value	Partial Eta Squared
1. Perceived knowledge to carry out supportive supervision	Intervention	3.55	4.64	GLM repeated measures	5.235	**0.034**	0.216
	Control	4.00	3.86				
							
2. Knowledge on how to do supportive supervision as a barrier	Intervention	3.55	1.73	GLM repeated measures	8.868	**0.008**	0.318
	Control	3.86	3.86				
							
3. Availability of resources to supervise immunization providers as a barrier	Intervention	4.00	2.73	GLM repeated measures	5.970	**0.024**	0.239
	Control	3.93	3.86				
							
4. Lack of clear format for providing supportive supervision as a barrier	Intervention	4.09	2.22	GLM repeated measures	6.173	**0.022**	0.245
	Control	3.71	3.21				
							
5. Lack of recognition of importance of supportive supervision by providers as a barrier	Intervention	3.64	1.91	GLM repeated measures	13.573	**0.002**	0.417
	Control	2.93	2.79				

*Regression models included: treatment group, age, years of experience in the current job, geographic area, and the interaction term "treatment group*geographic area".

Table 6 shows improvements in both the intervention and control districts for district level service delivery outcome indicators, but a greater improvement was observed in the intervention group. Results of univariate analysis (paired-sample t-test) indicate that in contrast to control districts, intervention districts significantly increased coverage rates for DPT-3 by 11.7% (P = 0.000), decreased contraindication rates by 1.93% (p = 0.057), decreased refusal rates by 1.47% (p = 0.044), and increased number of vaccinated children per 100 dose by five for DPT (p = 0.016), by six for OPV (p = 0.029), and by seven for HEP B vaccines (p = 0.022).

**Table 6:** Service delivery outcome indicators by treatment group and pre- and post-test.

	Mean %			
		
Service delivery outcome indicator 15 intervention and 15 control districts	Treatment group	Pre-test	Post-test	Paired Samples T test
1. DPT-3 coverage	Intervention	77.4%	89.1%	***P = 0.000***
	Control	81.3%	84.8%	*P = 0.371*
	*ANOVA*	*P = 0.294*	*P = 0.285*	
				
2. Polio-3 coverage	Intervention	64.1%	90.6%	***P = 0.000***
	Control	65.2%	82.2%	***P = 0.013***
	*ANOVA*	*P = 0.499*	*P = 0.173*	
				
3. Hep B-3 coverage	Intervention	62.9%	81.5%	***P = 0.002***
	Control	58.8%	68.1%	***P = 0.001***
	*ANOVA*	*P = 0.139*	*P = 0.172*	
				
4. Contraindications rate (mean for monthly contraindication rates to account for seasonal variation in contraindications)	Intervention	7.1%	5.2%	***P = 0.057***
	Control	5.1%	4.7%	*P = 0.432*
	*ANOVA*	*P = 0.160*	*P = 0.631*	
				
5. Refusal rate (mean for monthly refusal rates to account for seasonal variation in contraindications)	Intervention	5.9%	4.4%	***P = 0.044***
	Control	6.5%	5.0%	*P = 0.340*
	*ANOVA*	*P = 0.782*	*P = 0.606*	
				
6. Vaccine wastage DPT (calculated as number of vaccinated children per 100 dose)	Intervention	68	73	***P = 0.016***
	Control	68	66	*P = 0.387*
	*ANOVA*	*P = 0.936*	*P = 0.179*	
				
7. Vaccine wastage OPV (calculated as number of vaccinated children per 100 doses)	Intervention	65	71	***P = 0.029***
	Control	62	62	*P = 0.955*
	*ANOVA*	*P = 0.554*	*P = 0.036*	
				
8. Vaccine wastage HEPB (calculated as number of vaccinated children per 100 doses)	Intervention	73	80	***P = 0.022***
	Control	66	68	*P = 0.419*
	*ANOVA*	*P = 0.353*	*P = 0.125*	

When comparing the treatment groups in terms of the changes in district level outcome indicators from baseline to follow up (i.e. difference between groups for the "change magnitude") using ANOVA, it was found that the "change magnitude" was significantly higher in intervention group for the decrease in DPT vaccine wastage (p = 0.021), decrease in OPV vaccine wastage (borderline significance, p = 0.085), and for the increase in DPT-3 coverage (borderline significance, p = 0.075) (Table 7).

**Table 7:** Difference between treatment groups in the magnitude of change from baseline to follow up in the service outcomes (ANOVA).

	Mean %	
	
Service delivery outcome indicator 15 intervention and 15 control districts	Treatment group	Magnitude of change
9. DPT-3 coverage	Intervention	11.7%
	Control	3.6%
	*ANOVA*	***P = 0.075***
		
10. Polio-3 coverage	Intervention	26.5%
	Control	17.1%
	*ANOVA*	*P = 0.159*
		
11. HepB-3 coverage	Intervention	12.5%
	Control	15.3%
	*ANOVA*	*P = 0.586*
		
12. Contraindications rate (mean for monthly contraindication rates to account for seasonal variation in contraindications)	Intervention	-1.9%
	Control	-0.5%
	*ANOVA*	*P = 0.192*
		
13. Refusal rate (mean for monthly refusal rates to account for seasonal variation in contraindications)	Intervention	-1.5%
	Control	-1.5%
	*ANOVA*	*P = 1.000*
		
14. Vaccine wastage DPT (calculated as number of vaccinated children per 100 dose)	Intervention	4.9
	Control	-1.9
	*ANOVA*	***P = 0.021***
		
15. Vaccine wastage OPV (calculated as number of vaccinated children per 100 doses)	Intervention	0.6
	Control	-0.1
	*ANOVA*	***P = 0.085***
		
16. Vaccine wastage HEP B (calculated as number of vaccinated children per 100 doses)	Intervention	6.8
	Control	1.9
	*ANOVA*	*P = 0.178*

The results of the FGDs of CPH and health care facility staff in intervention districts point to a number of improvements from baseline to follow up. These included: an improved management/supervision approach from punitive to supportive; improved knowledge of providers about contraindications to immunization; better clarification of the roles and responsibilities of staff at district CPH and facility level; an increased sense of job responsibility regarding roles in immunization program; and an increased ability of CPH staff to carry out supportive supervision.

**Figure 1:**
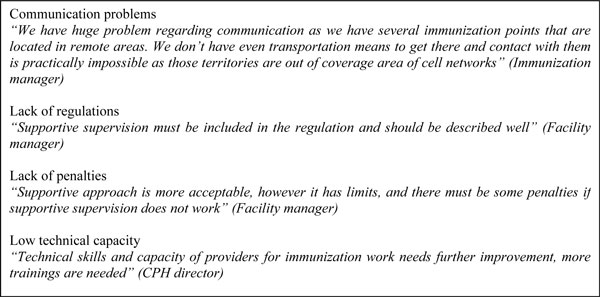
**Quotations from focus group discussions regarding health system barriers**.

Despite aforementioned improvements, the FGDs highlighted several potential barriers that remained over the course of intervention and hinder the implementation of the intervention and the use of certain tools to their fullest extent (Figure 1). The barriers mentioned included: communication problems with remote health facilities; lack of official regulations on supportive supervision; lack of CPH authority to impose penalties on low-performing health facilities; low technical capacity of local health providers; and inability to give financial incentives to well performing facilities and providers.

## Discussion

The results of the study suggest that the intervention package, which included supportive supervision guidelines, district-level trainings, continuous supervision and support during a 12 month period, monitoring and evaluation of provider performance and funding for district CPH to carry out the supportive supervision missions (travel and communication costs), were implemented as planned. As for the number of expected improvements among immunization managers, the intervention independently contributed to improved knowledge of supportive supervision, and helped remove self-perceived barriers to supportive supervision such as availability of resources to supervisors, lack of clear format for providing supportive supervision, and lack of recognition among providers on the importance of supportive supervision.

Similarly, the results of the analysis show that improvements were recorded in both the intervention and control districts for district level service delivery outcome indicators; however, a greater improvement was observed in the intervention group. This latter observation can be attributed to the intervention package that independently contributed to improved service outcomes, namely, decreasing vaccine wastage and increasing immunization coverage. The obvious trend in improvement of service outcomes in both intervention and control districts can be attributed to other factors such as an overall improvement in health care financing and targeted service provision to the poor that took place in the country over the course of intervention [16]. Positive country-level economic growth may have also contributed to the improved population access to health care, [17] resulting in the improved immunization coverage rates. Prior to implementation of our study in 2004, a considerable proportion of the Georgian population faced financial access barriers to health care [18]. Furthermore, interventions aimed at increasing access to services may improve performance of the immunization program [19]. The health care financing initiatives of the Government of Georgia certainly helped improve access to services for the population and most importantly for the poor [16]. Therefore, it is possible that improved access to health care may have contributed to improved immunization coverage rates in Georgia.

An equal but modest decrease in the rate of refusals to vaccination (as declared by parents) in both intervention and control districts was found. A Georgian study conducted in 2002 showed that the community members in Georgia had little knowledge of Vaccine Preventable Diseases (VPD); in particular, there was inadequate knowledge of how VPDs are transmitted, of the complications of VPDs as well as inadequate knowledge regarding the safety of immunization and the quality of vaccines [20]. It has been shown that strategies to increase demand through improving knowledge among clients regarding need for vaccination are useful [19]. It is possible that some improvement in community members' knowledge of VPDs in both intervention and control districts occurred, however this was not assessed in our study.

### Limitations

The results should be cautiously interpreted given the limitations of this study. First, most individual level data are subjective and social desirability bias may have confounded the results one year after the intervention. Second, there is the possibility that a modest role of intervention in improving service outcomes could be due to a type 2 error, resulting from inadequate number of districts (budgetary limitations would not allow to expand intervention to more districts) to ensure enough power of the study to ascertain greater effect of the intervention in the observed changes in the service outcomes. Finally, the short duration of the intervention may have restricted the intervention's potential to bring the expected outcomes.

## Conclusion

Provider-based interventions such as supportive supervision can have independent positive effects on immunization program indicators. Thus, it is recommended to implement supportive supervision within the framework of national immunization program in Georgia and other countries in transition with similar institutional arrangements for health services organization.

## List of abbreviations used

ANOVA: Analysis of variance; CPH: Centre of public health; FGDs: Focus group discussions; GLM: Generalized linear model; MIS: Management information system; NIP: National Immunization Program; VPD: Vaccine preventable disease.

## Competing interests

The authors declare that they have no competing interests.

## Authors' contributions

The authors contributed to the conception, design, and interpretation of the study. MD conceived, drafted and finalized the manuscript. GG and AZ contributed to the conception of the manuscript and drafting the manuscript.

MD, GG and AZ contributed to the implementation of study in Georgia. GM, LE and JCK conducted review and provided comments on the manuscript.

## Supplementary Material

Additional file 1 Abstract in Russian. Click here for file
