# Supplementary material for: The role of supportive supervision on immunization program outcome - a randomized field trial from Georgia
Source: BMC Int Health Hum Rights. 2009 Oct 14;9(Suppl 1):S11. doi: 10.1186/1472-698X-9-S1-S11 (PMC3226230; doi:10.1186/1472-698X-9-S1-S11)
Supplement: Additional file 1 — Abstract in Russian. [file 1472-698X-9-S1-S11-S1.pdf]

Роль Поддерживающей Супервизии для Результатов Программы Иммунизации – Рандомизированное Полевое Исследование в Грузии

Мамука Джибути, Георгий Готсадзе, Акаки Зоидзе, Георгий Матарадзе, Лора Исмеил, Джилиан Клеар Кохлер

Абстракт

Обоснование

Одним из основных барьеров для улучшения степени охвата иммунизацией, является человеческие ресурсы и менеджмент человеческих ресурсов. В Республике Грузия, стране где в последней декаде проводились широкомасштабные реформы здравоохранения, недавно была проведена интервенция с целью улучшения осуществления програмы иммунизации. Были проведены ряд измерений для удостоверения в том, что менеджеры иммунизации эффективно осуществляют свою активность посредством прямого, персонального регулярного контакта, для руководства, поддержки и помощи персоналу выбранного учреждения здравоохранения, с целью увеличить их компетентность в работе по иммунизации. Целью этого исследования было документировать эффективность «поддерживающей» супервизии для выполнения программ иммунизации на уровне районов Грузии.

Методы

Был использован дизайн пре-пост экспериментального исследования для количественной оценки. Данные брались из базисного и последующих опросов работников здравоохранения и менеджеров иммунизации 15 интервенционных и 15 контрольных районов. Эти данные дополнялись дискуссией фокус-группы Центра Общественного Здравоохранения и персонала учреждления здравоохранения.

Результаты

Результаты исследования показывают, что пакет интервенции вызвал ряд ожидаемых улучшений. Среди менеджеров иммунизаци, интервенция независимо содействовала улучшению знания поддерживающей супервизии и помогла удалению барьеров для самосовершенствования, таких как доступность ресурсов для супервайзера, отсутствие понимания работниками зхдравоохранения важности поддерживающей супервизии. Интервенция независимо способствовала относительным улучшениям результатов предоставления сервисов на уровне районов, таких как показатели потерь вакцины и степень охвата иммунизацией DPT-3. Отчетливый положительный сдвиг в результатах предоставления всех сервисов как в интервенционных, так и контрольных районов, может быть отнесен за счет общего улучшения доступности здравоохранения для населения в Грузии.

Выводы
Провайдерские интервенции, такие как поддерживающая супервизия, могут иметь независимый положительный эффект на индикаторы программ иммунизации. Следовательно, рекомендуется имплементировать поддерживающую супервизию в рамки национальных программ иммунизации в Грузии и других странах переходного периода с похожим институциональным устройством системы здравоохранения.
